# Nursery Government in Its Sanitary Aspects

**Published:** 1857-01

**Authors:** T. Herbert Barker


### NURSERY GOVERNMENT IN ITS SANITARY ASPECTS.

By T. HERBERT BARKER, M.D., F.R.C.S.

(*Continued from* p. 152.)

*Injurious Effects of Narcotics.* When the rules of infantine health are disregarded by mothers and nurses; when food wrong in quality or excessive in quantity is habitually given; when cleanliness, sufficient repose, gentle exercise and pure air have been neglected, we must expect the infant constitution to suffer. Irritation, wakefulness, a bloated or emaciated habit of body and peevishness of temper will probably appear as the results of such mismanagement. And now, as one error leads to another, the inexperienced mother or nurse, having first produced disease, proceeds to exasperate that disease by the most mischievous quackery—in short by "drugging" the infant. Its cries are distressing, it will not sleep; it is evidently suffering pain;—the pain must be allayed—the child must be put to sleep;—but what are the means to be used? Nature calls loudly for help and receives—poison! An ignorant neighbour informs the distressed mother of the wonderful virtues of a certain elixir—"Godfrey's Cordial", "Dalby's Carminative", "Poppy tea", "Diocodium and peppermint", or some other cloak for opium. In one respect these destructive nostrums fulfil their promise. The cries of the child are effectually "stilled"; for, in many cases, he is soon silent—in the grave! Let it not be thought that we write too strongly of this murderous practice. I have seen even in the course of my own experience in a rural district, too many instances of the injurious effects of narcotics upon children; but it is in the manufacturing districts that the practice of "drugging" is carried on in a wholesale manner. On this sad topic the Registrar-General has written as follows:

"How pitiful is the condition of many thousands of children born into the world! Here, in the most advanced nation in Europe, in one of the largest towns of England,—in the midst of a population unmatched for its energy, industry and manufacturing skill—in Manchester—the centre of victorious agitation for commercial freedom—aspiring to literary culture—where Percival wrote and Dalton lived—thirteen thousand three hundred and sixty-two children perished in seven years, over and above the mortality natural to mankind! These 'little children,' brought up in unclean dwellings and impure streets, were left alone, long days, by their mothers to breathe the subtle sickly vapours—soothed by opium, a more 'cursed' distillation than 'hebenon,' and when assailed by mortal diseases—their stomachs torn, their bodies convulsed, their brains bewildered, left to die without medical aid which, like hope, should 'come to all'—the skilled medical man never being called in at all, or only summoned to witness the death and sanction the funeral!"*

Such remarks, I trust, are only required by the most ignorant mothers and nurses in the lowest grades of society. Yet I beg leave to intimate that such drugs as those referred to, may sometimes be found in the possession of nurses in the higher classes of society. The mother who wishes her infant to grow up with "a sound mind in a healthy body", cannot guard too strictly against the use of poisons.

*Diet during the Second Stage of Infant Life.* I have now to treat of the second stage of infancy, namely, of that beyond the sixth or seventh month, in which other articles of food may be added to the milk-diet. But let it be observed that a child will require a *gradual* weaning from its early artificial diet, as from the breast. The first deviation from a purely liquid diet may commence in the seventh or eighth month, and may consist of a little soft bread, steeped in hot water, with the addition of fresh cow's milk and a small quantity of sugar. After this has been used for some time, some light broth may be given to vary the bill of fare; but this must be free from fat and vegetable matter.

Variations of diet may be required during this second stage of infancy; but the rule of simplicity, lightness, and

---

* Ninth Annual Report of the Registrar-General of Births, Deaths, and Marriages in England. 1849.

digestibility should always be observed. A diet which agrees well with one child will sometimes be found to disagree with another. Thus prepared barley dressed with water and unboiled milk will in some cases, where there is a tendency to constipation of the bowels, be found to agree well; in others it will prove too laxative. This may sometimes be obviated by boiling the milk. Or, in this case, genuine arrow-root (well cooked) may be useful. *But such articles as arrow-root, sago, or tapioca, should never be wholly depended upon as constituents of the diet of infants;* for they are deficient in some of the requisite elements of nutrition. If the infant suffer much from flatulence, it is advisable to boil a few caraway seeds in water, and carefully strain it before mixing it with the food.

The simplest deviation from the more liquid diet to which the infant had been accustomed is the bread and milk as recommended above, and this is probably the form of food liable to the least risk of error in the mode of its preparation, and should be persevered with if it is found to agree well with the infant. In order, however, to meet the requirements of different cases, a list of preparations is subjoined in the foot-note, from which it will not be difficult to make a selection suitable to almost every case.* Bear in mind this

---

* 1. *Bouillie* (a French preparation), commonly known in this country as *baked flour* food, may be safely recommended to mothers, as well worthy of a trial. It is made by roasting very gently the best wheat-flour in a slow oven, and afterwards boiling, or rather simmering it for a considerable time, either in water or milk and water, then adding a little sugar. When it is well made, it should be free from knots or lumps, and not too thick.

2. If the above should not agree with the infant (although it often does, if properly made) the *boiled flour* food may be tried. Take a pound of flour, put it in a cloth, tie it up tightly, then put it in a saucepanful of water, and let it boil four or five hours; then take it out, peel off the outer rind, and the inside will be found quite dry, which grate. A small quantity of this boiled flour should be made into food in the same way as gruel is made, and then slightly sweetened with lump sugar. New milk, provided it agree with the child, may be added to this preparation.

3. A French spoon-food, called *crême de pain*, may be prepared according to the following simple recipe:—Take a few slices of well-baked bread, and dry them well (but do not burn them) in an oven; then infuse them in water for several hours, and let them simmer for a considerable time, adding now and then a little more water, that the sop may not become too thick. Sweeten it moderately, and add (if you please) a few drops of orange-flower water.

4. The *lait de poule* has been referred to.

5. The farinaceous food for infants prepared by Hards of Dartford, Dodson's biscuit powder, or Lemann's preparations, may sometimes be used with advantage.

6. Bullock's semola, before referred to, is an excellent preparation, of very uniform strength, and has been found by the writer, when properly cooked according to the printed directions accompanying it, to agree remarkably well with the digestive organs of the infant. This preparation is rich in gluten, rule:—*When you have found a diet which evidently agrees well with the child's constitution, do not, for the sake of change or to try a mere experiment, make alterations in that diet.*

Whatever the kind of food may be, let it always be prepared immediately before use, and let all the vessels used in cooking be kept perfectly clean. The food should be given to the infant at a tepid or lukewarm temperature. Until the infant is old enough to take the thicker kind of milk-sop, preference should be given to the bottle, rather than to the spoon or to the boat. The bottle should be made of colourless glass, and the form we prefer is made without the opening in the centre, and with a wide opening at one end, into which a large cork, with a well-made ivory mouthpiece piercing its centre, is fitted. I believe that this is the most convenient and cleanly apparatus.* Great care should be taken to keep it very clean, or the particles of diet adhering to the inside will ferment and produce acidity. To avoid this, the most convenient way is to use two bottles, so that one may be thoroughly well cleansed, while the other is in use. Never put a second supply of food upon the remains of a former, unless a very short interval has elapsed, and they are of the same making. The tube must be kept clean;

---

the pure nutritive or flesh-making principle of wheat. One part is equal in nutritive power to five parts of wheaten flour, and it is as digestible as it is nutritious. The manufacturer's well known chemical attainments have been usefully exercised in the preparation of a really valuable article of diet, both for the infant and for the invalid.

7. The *rusk food* is very useful in some cases, and may be made with rusks, boiled for an hour with water, which should then be either strained through a sieve, or well beaten up by means of a fork, and slightly sweetened with lump sugar. Great care should be taken to select good rusks, as few articles vary so much in quality.

8. Another useful food is the top crust of a baker's loaf, boiled for an hour with water, and then moderately sweetened with lump sugar. If at any time the child's bowels should be costive, *raw* may be substituted for *white* sugar, in any of these preparations; and should *moist* sugar not answer the purpose, a small lump of *manna* may be used instead.

9. *Rice food* may be prepared in the following manner:—Soak some *best* rice in cold water for an hour; strain, and add fresh water to the rice; then let it simmer till it will pulp through a sieve: put the pulp and water into a saucepan with a lump or two of sugar, and again let it simmer for a quarter of an hour. A portion of this may be mixed with new milk, so as to make it of the thickness of cream, and should be given by means of the bottle. If the bowels are much relaxed, the milk may be boiled, but not otherwise.

10. The following is a good food, when an infant's bowels are weak and relaxed:—Into five large spoonfuls of the purest water, rub smooth one dessert-spoonful of fine flour. Set over the fire five spoonfuls of new milk, and put two lumps of sugar into it; the moment it boils, pour it into the flour and water, and stir it over a slow fire twenty minutes.

if an artificial teat be used, very great care must be taken to keep it sweet, and in the intervals between use it should be kept in a mixture of gin or whiskey and water.

*Diet during Childhood.* The following bill of fare may be regarded as generally sufficient for a child of two or three years. On awaking early in the morning, a little bread and milk may be given, or (while the child is too young to eat solid bread), a sop of bread in warm milk. The child will then generally sleep again for an hour or two. A second meal may consist of bread softened in hot water. The water being drained off, milk and sugar may be added. This may be taken about nine o'clock. The early meal will probably be dispensed with, if the wholesome practice of putting the child to bed early in the evening is not pursued—but if the mother wishes to rear a healthy progeny, she will by no means neglect this important point. Between one and two o'clock, or in the general dinner hour, a little broth made of the lean part of beef or mutton, or chicken-broth, with a slice of bread, will make an excellent meal. When a sufficient number of teeth show that the child is able to masticate solid animal food, a little beef or mutton plainly roasted or boiled, with such fresh vegetables as potatoes, turnips, and cauliflowers, thoroughly cooked, may be given. Until thorough mastication of the solid animal food can be performed by the teeth, it must be finely divided, in fact minced, or the child will suffer from disorder of the digestive organs and innutrition. The mother must not be satisfied with giving directions on this point to the nurse, but must see that it is properly attended to. Accustom the child to eat its food slowly, and to drink some time *after* dinner. Copious draughts during the time of eating should be avoided. This was a rule laid down by Abernethy in reference to the diet of adults, and the habit should be commenced during childhood. The best beverage for children is toast-water, freshly made. The latest meal—bread and milk—should be taken at six o'clock in the evening, not later. Soon afterwards, the child should go to bed. In the fourth or fifth year, the bread and milk may be given without water. At this age also the early meal on awaking in the morning may be discontinued.

This course of food will not suit all stomachs. Meat or broth every day would perhaps lead to fulness of the system in some. But it will be easy to observe this, and accordingly to lighten the quality and the quantity of the diet. A lightly boiled egg may occasionally be substituted with advantage for meat. *Cocoa* is a more suitable beverage for children than tea. Ripe fruits, such as the orange, strawberries, currants, a few grapes the skins being rejected, and roasted apples, may be allowed; but stone-fruits and nuts must be avoided, also dried fruits, with the exception of figs. Whatever variations may be made, let the whole course of diet be simple, bland, and nutritious. Avoid pastry, pork, veal, salt-beef, new or heavy bread, tea-cakes, strong tea, sweetmeats, and especially (the importance of this point will bear repetition) all alcoholic beverages. That mother will show sound wisdom who keeps her children as long as possible ignorant even of the taste of ale, wine, and spirits.

To conclude; the principal rules already explained may be here recapitulated in brief terms. 1. In all normal cases, or, in other words, in all cases where no insurmountable difficulty or objection exists—the infant should be nourished in nature's own inimitable mode—*by the mother*. In this way only can we give the highest human security for the preservation of infant life and health. 2. Avoid the common error of administering medicine or indigestible food to the infant soon after its birth. 3. When the mother is not able to nurse, let the nearest and best substitute for nature's provision be found. Let the infant be committed to the care of a healthy and suitable wet-nurse. 4. In cases where it is certain that the infant can be nourished by the breast only for a very short period (say, a few weeks), and where a suitable wet-nurse cannot be engaged, it is better to give no nutriment from the breast, but, at once, to begin with the best artificial diet. 5. The process of *weaning* should be gradual, and great care must be exercised in the choice of food, according to the rules already given. 6. The artificial food of early infancy must be, in form, consistence, and quality, the nearest possible imitation of the mother's milk. 7. Give no solid food until the teeth have appeared. 8. Never depend on such articles as sago, arrow-root, or tapioca, as *main* ingredients of infantine diet. 9. The change from a liquid to a rather solid form of diet must be gradually and cautiously made. 10. Never allow either narcotics or alcoholic stimulants (*in any form whatever*) to be administered to infants or children, excepting under medical direction and care.

(*To be continued.*)

